# Learning to Identify Severe Maternal Morbidity from Electronic Health Records

**DOI:** 10.3233/SHTI190200

**Published:** 2019-08-21

**Authors:** Cheng Gao, Sarah Osmundson, Xiaowei Yan, Digna Velez Edwards, Bradley A. Malin, You Chen

**Affiliations:** a Department of Biomedical Informatics, Vanderbilt University Medical Center, Nashville, TN, United States; b Department of Obstetrics and Gynecology, Vanderbilt University Medical Center, Nashville, TN, United States; c Sutter Research, Development and Dissemination, Sacramento, CA, United States; d Department of Biostatistics, Vanderbilt University Medical Center, Nashville, TN, United States

**Keywords:** Electronic health records, machine learning, severe maternal morbidity

## Abstract

Severe maternal morbidity (SMM) is broadly defined as significant complications in pregnancy that have an adverse effect on women’s health. Identifying women who experience SMM and reviewing their obstetric care can assist healthcare organizations in recognizing risk factors and best practices for management. Various definitions of SMM have been posited, but there is no consensus. Existing definitions are further limited in that they 1) are often rooted in existing clinical knowledge (which is problematic as many risk factors remain unknown), leading to poor positive predictive performance (PPV), and 2) have limited scalability as they often require substantial chart review. Thus, in this paper, a machine learning framework was introduced to automatically identify SMM and relevant risk factors from electronic health records (EHRs). We evaluated this framework with EHR data from 45,858 deliveries at a large academic medical center. The framework outperformed a state-of-the-art model from the U.S. Centers for Disease Control and Prevention (AUC of 0.94 vs. 0.80). Specially, it improved upon PPV by 59% (CDC: 0.22 vs. our model: 0.35). In the process, we revealed several novel SMM indicators, including disorders of fluid or electrolytes, systemic inflammatory response syndrome, and acidosis.

## Introduction

Severe maternal morbidity (SMM) is a physical condition that results from, or is aggravated by, pregnancy and has an adverse effect on a woman’s health. [1] SMM is an umbrella for various phenomena, including but not limited to, cardiomyopathy, hemorrhage, organ failure, pregnancy-induced hypertension and embolism, pulmonary embolism sepsis, seizure shock, severe amniotic fluid embolism, and uterine rupture. SMM can be considered a near miss for maternal mortality because, without in-time identification and treatment, these conditions can lead to maternal death [2; 3]. Thus, SMM is utilized as a new indicator of the quality of maternal health. [4] Its prevalence has steadily increased in low- and middle-income countries, as well as the developed world. [4–6] It now affects around 60,000 women in the U.S each year. [6] At the same time, the U.S. Department of Health Resource and Services Administration has increasingly focused on reducing the rate of SMM and improving its survival rate [7].

Identifying women who experience SMM and reviewing their obstetric charts can enable clinicians and healthcare organizations, more generally, to better understand the primary etiologies and contributing factors of SMM. In doing so, this can lead to a refinement of care management practices to prevent SMM or improve the quality of its management. However, there are major challenges in identifying SMM, which partially arise due to the lack of a consensus definition. For instance, the Centers for Disease Control and Prevention (CDC) has provided a definition based on 25 SMM indicators in the form of diagnosis and procedure codes [1]. The CDC definition is straightforward to specify and, thus, easy to implement in that International Classification of Disease (ICD) codes are readily available in hospital discharge data. However, it does not indicate the severity of maternal morbidity and, as a result, its application leads to many false positives and negatives. [8]

Another popular definition of SMM has been proposed by experts from the American College of Obstetricians and Gynecologists (ACOG) and the Society for Maternal-Fetal Medicine (SMFM). This definition is a guideline that allows for each birthing facility to determine their own SMM criteria [8; 9]. The guidelines are based on two primary factors: i) admission to an intensive care unit (ICU) or ii) transfusion of four or more units of blood. [7] These may be supplemented by other factors, such as intubation for at least 12 hours, organ system failure, and unanticipated surgical intervention [8]. Although SMM identification approaches built upon the guidelines can achieve high levels of performance [8], they have several deficiencies. First, the approach designed for one facility cannot be directly applied by another facility. This is because the risk factors used are often facility-specific and are unique unto a facility. [6] Second, the process of SMM identification requires substantial manual effort in the form of in-depth chart reviews, which incurs a nontrivial amount of time and effort.

Recent studies have shown that machine learning algorithms along with data in electronic health records (EHR) can be leveraged to identify or predict a variety of diseases [10–11]. For example, supervised machine learning algorithms were applied to identify different types of cancers and their progression pathways [11]. Another example [10] is the application of deep learning algorithms to predict Alzheimer’s disease in its early stage. To identify SMM during delivery hospitalizations, we introduce a machine learning framework that incorporates data in EHRs. This framework has several notable benefits in that 1) it leverages standardized clinical terminologies as inputs, and thus models developed under the framework can be applied in EHRs of other facilities; 2) it can deal with over a thousand clinical concepts with reduced manual effort; and 3) it allows for the discovery of novel factors to identify SMM.

## Methods

**Figure 1–:**
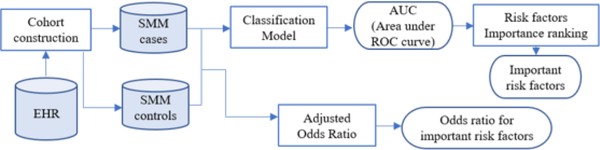
Data workflow

We begin this section with a description of the data workflow as shown in Figure 1. First, before feeding data into the classification model, we annotate EHRs as SMM cases and controls. The classification model is then evaluated in terms of area under the receiver operating characteristic (ROC) curve (AUC). Lastly, risk factors are ranked according to odds ratios and their contributions to the performance of the classification models.

### Dataset

We collected 13 years of data (2005 to 2018) from the Vanderbilt University Medical Center (VUMC) EHR system, which includes 45,858 deliveries for obstetric inpatients. This dataset includes patient demographics (e.g., age and race), admission notes, discharge summaries, and clinical concepts in the form of diagnoses (ICDs) and procedures (CPTs). A summary of the medical concepts and demographics are provided in Tables 1 and Table 2, respectively. We note that the diagnosis and procedure codes were transformed into standard terminologies defined under the Observational Medical Outcomes Partnership (OMOP) common data model, operated by the Observational Health Data Sciences and Informatics (OHDSI) consortia [12]. All these codes in this study are called medical concepts.

In Table 1, it can be seen that deliveries with SMM have more assigned diagnoses and procedures than those without SMM. This implies that deliveries with SMM are more complicated. There was a total 3,972 unique diagnosis concepts and 1,581 unique procedural concepts. In Table 2, it can be seen that there was a greater percentage of black women experiencing SMM (24%) than those without (18.5%).

### Cohort construction

We use the criteria *four or more units of blood transfusion or ICU admission* developed by the ACOG to identify SMM candidates [8]. Using this criterion, 594 SMM candidates were identified. Obstetricians then reviewed the charts for these candidates and excluded 182 candidates who did not experience SMM. Our final cohort contained 412 SMM cases (0.9% of all deliveries). We assume that all of the remaining deliveries were controls. We acknowledge that the controls may contain a small number of SMM cases that cannot be captured by the ACOG criteria. A recent study [8] shows the number of missed cases is likely too small to impact the performance of identification models.

### SMM identification models

#### Feature engineering

For each instance, we extract their assigned diagnoses and procedures represented by medical concepts using the OMOP common data model. Each medical concept is treated as a binary feature, where 1 indicates a patient was assigned the concept during the delivery and 0 otherwise.

Since the number of diagnoses (3,972) and procedures (1,581) is substantially larger than the number of positive instances, which may influence the performances of machine learning models. As such, we aim to do feature selection before applying machine learning models. To orient the system to be more generalizable, we adopt a filter feature selection algorithm without using class information (i.e., SMM vs. Non-SMM) and machine learning models. The feature selection algorithm ranks the concepts according to their frequency occurring in the investigated patient population. We incrementally add top ranking features into the machine learning models. Specifically, we train models using the top 10%, 20%, 40%, 80% and 100% of the features

#### Balanced versus unbalanced cohorts

As noted earlier, the dataset is highly imbalanced - less than 1% of the women experienced SMM. As such, learning models based on the observed case to control ratio could lead to bias in the model. To mitigate this problem, we randomly sample controls (Non-SMM) from the data to investigate case:control ratios in the form of 1:1, 1:4, 1:16 and 1:64. In addition, we evaluated how the ratio impacts the performance of machine learning models to identify SMM from the natural case:control ratio.

#### Regularized logistic regression models

##### We train four types of models

*M*_0_: 〈CDC-25, no learning〉 The CDC model, which leverages 25 indicators to identify SMM. This model is considered as the baseline.

*M*_1_: 〈diagnoses, ridge logistic regression〉 The features are diagnosis concepts and ridge logistic regression (*L*_2_) is used as machine learning models. Under this category, we train regression models using top 10%, 20%, 40%, 80% and 100% of the diagnosis codes, and datasets with case:control ratios as 1:1, 1:4, 1:16 and 1:64.

*M*_2_: 〈procedures, ridge logistic regression〉 The features are procedural concepts and ridge logistic regression is used as machine learning models. We use the same strategy in *M*_1_ to train the regression models.

*M*_3_: 〈diagnoses and procedures, ridge logistic regression〉 The features are diagnosis and procedural concepts and ridge logistic regression is used as machine learning models. We use the same strategy in *M*_1_ to train regression models.

For each model, we apply 10-fold stratified cross-validation. The performance of models is measured in terms of sensitivity (SN), specificity (SP), positive predictive value (PPV), and AUC.

#### Medical concept ranking algorithm

To determine which medical concepts play more important roles in identifying SMM, we use the following equation to rank concepts:

$$F_{imp}^{j}=\sum_{i=1}^{10}\frac{AUC_{i}}{\sum_{l=1}^{10}AUC_{i}}\beta_{i}^{j},$$

where $F_{imp}^{j}$ is the overall importance of the *j*^*th*^ concept and $\beta_{i}^{j}$ is the coefficient of the *j*^*th*^ concept for the *i*^*th*^ cross-validation model. The medical concept importance is adjusted by the model performance (AUC) and the importance of a concept within a model (coefficient). The larger the value of $F_{imp}^{j}$ is, the more important the *j*^*th*^ concept is. For instance, imagine we train two models *m*_1_ and *m*_2_ on two medical concepts *c*_1_ and *c*_2_, such that the AUC of the models was 0.8 and 0.9, respectively. It was found that the coefficients of the two concepts in *m*_1_ and *m*_2_ was (0.6, 0.4) and (0.8, 0.2) respectively. According to our approach, the importance of medical concept *c*_1_ is (0.8 / (0.8 + 0.9)) × 0.6 + (0.9 / (0.8 + 0.9)) × 0.8, or 0.71.

We learn a logistic regression model based on the medical concepts with the largest importance scores, along with age and race. Odds ratios are calculated for each feature in the SMM and non-SMM classes along with their corresponding *p*-values. The OR was adjusted for age and race and the *p*-value was subject to a Bonferroni correction.

## Results

### Influence of the number of features

**Figure 2–:**
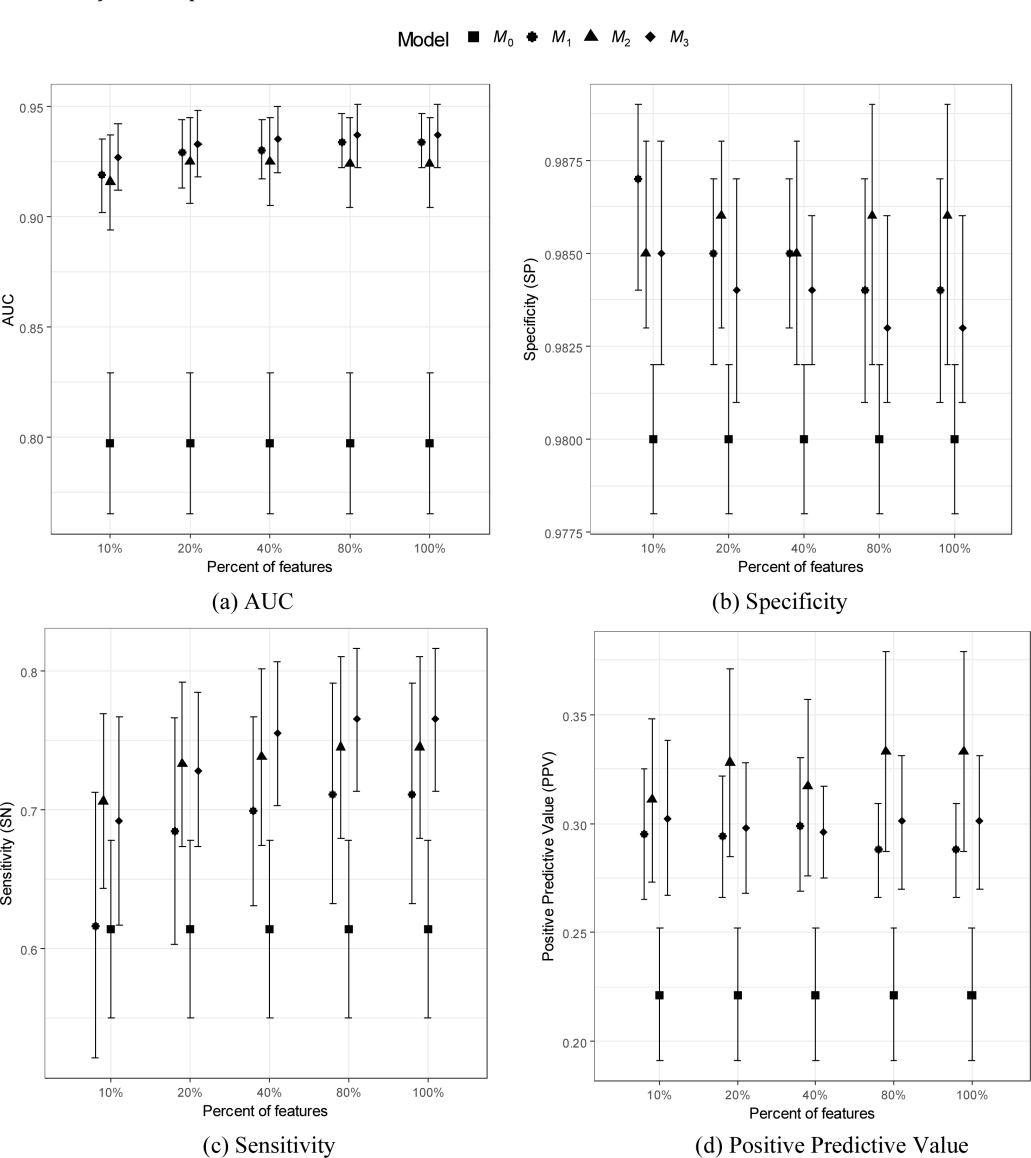
SMM recognition performance as a function of the number of features in the logistic regression model

We use the natural proportion of cases to controls to train and validate models using the top 10%, 20%, 40%, 80% and 100% of concepts. The performance (in terms of AUC, SN, SP, and PPV and the corresponding 95% confidence interval) is depicted in Figure 2.

In Figure 2(a), it can be seen that *M*_1_, *M*_2_, and *M*_3_ outperform the baseline *M*_0_. Their performance improves as more features are added into these models, with an increase in AUC from 0.919 to 0.937. At each percentage of features, model *M*_3_ exhibits the best performance of all three models. Model *M*_3_ with 80% of features achieves the best AUC of 0.937, which is 18.6% better than *M*_0_ (0.790).

Figures 2(b) and 2(c) show the specificity and sensitivity with respect to the percentage of features considered. It is observed that the best specificity is achieved at 10% of the features for *M*_1_, improving by about 1% over the baseline model *M*_0_, while the sensitivity improves by 25% from 0.614 for *M*_0_ to 0.765 for *M*_3_ (which occurs when 80% of the features are used). Figure 2(d) shows the PPV for the models. The PPV clearly improves through *M*_1_, *M*_2_, and *M*_3_, where *M*_2_ has the best PPV 0.347 (which occurs when 80% of the features are used), which is 57% better than that achieved by the baseline *M*_0_.

### Influence of the case:control ratio

**Figure 3–:**
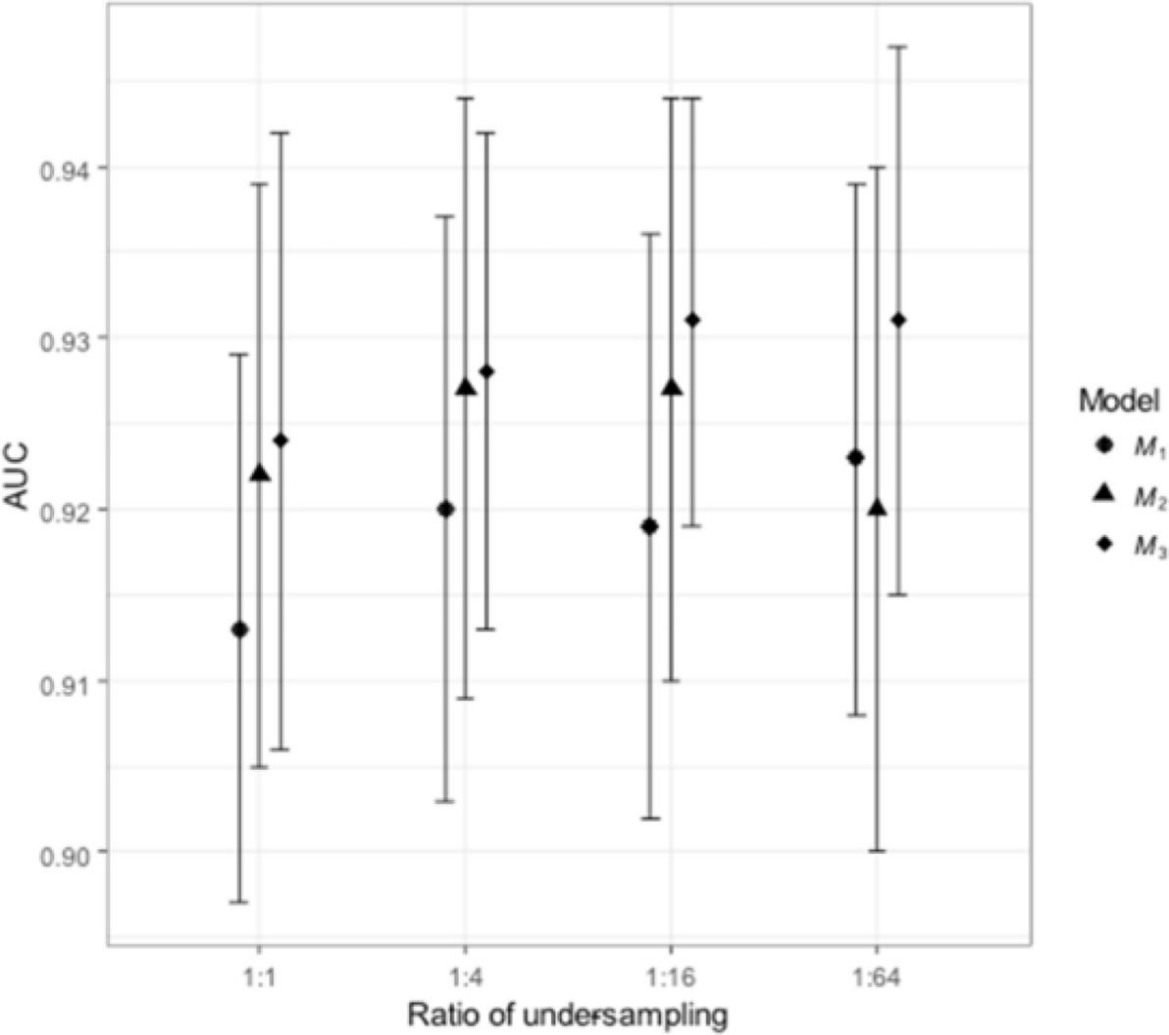
AUC performance as a function of SMM case:control ratio

Figure 3 shows the identification performance under different case:control ratios with all the features. It is notable that the performance does not always improve as the sampling ratio increases. *M*_1_ and *M*_3_ achieve the best AUC for the 1:64 sampling ratio while *M*_2_ achieves the best AUC at 1:4.

### Important concepts

We rank features using *M*_3_ with 80% of concepts because it exhibits the best AUC under a natural ratio of cases and controls. Out of 5,553 concepts, the top 10 positive concepts are reported in Table 3. The ranks of these features are reported, along with their concept names and odds ratios.

The concepts in Table 3 can be categorized into three groups. The first group is respiratory-related, including the 4^th^ concept. This has face value as patients with acute respiratory failure may need to be intubated or are dependent on ventilation. This leads to the second group, which includes the 1^st^, 2^nd^, and 5^th^ concepts. These concepts indicate that most of the patients who are intubated or need ventilation assistance experience SMM. The procedural concept “*Critical care, evaluation and management of the critically ill or critically injured patient*” (3^rd^ concept) is positively associated with SMM.

In addition, three novel risk factors (7^th^, 8^th^, and 9^th^ concepts) were found to be associated with SMM in this study. Most of the patients that were assigned these concepts experienced SMM. To the best of our knowledge, these concepts have not been reported in the literature nor are they in the CDC definition.

## Discussion and Conclusions

SMM often leads to adverse outcomes, including prolonged length of stay and an increased rate of postpartum readmission. The machine learning-based framework introduced in this paper can enable the automatic identification of SMM events, based on diagnosis, procedural and demographic features, and provide intuition into risk factors associated with SMM. In particular, this work has two notable findings.

First, the framework shows that the machine learning approach outperforms the baseline model (CDC criteria) in terms of AUC, positive predictive value and sensitivity. As such, we believe that a greater number of SMM cases can be identified through our framework, with a smaller false positive rate, than that is currently achieved through current best practice. This should substantially reduce the amount of manual effort to identify SMM instances. In addition, since we rely on the OMOP common data model, the proposed framework is likely to be generalized to other institutions.

Second, our model uncover potentially novel concepts that are positively associated with SMM. 85% (23 of 27) patients diagnosed with fluid and/or electrolytes disorder experienced SMM during their delivery hospitalization. Similarly, 82% (23 of 28) patients diagnosed with systemic inflammatory response syndrome developed SMM.

Despite these findings, it should be recognized that this is a pilot study and there are several limitations that need to be addressed as this line of research moves forward.

First, we assumed that our constructed control group does not contain any SMM cases; however, this is not guaranteed. It is challenging to solve such a limitation in a pilot study. This is because it would require substantial effort to review all 45,446 control instances to identify missed SMM cases. A recent study indicates that there may exist 1 SMM cases per 10,000 samples in the control group [9]. Still, given to the small missed-case:control ratio (1:10000), this situation likely has little impact on the performance of the SMM identification models.

Second, we only applied the simplest machine learning algorithm-logistic regression in this study and many other advanced machine learning algorithms are missing. We anticipate adding neural networks, decision tree, support vector machine (SVM), and gradient boosting classifier in our next-step study to improve SMM identification performances.

Third, more advanced feature selection algorithms can be used to preselect candidate features feeding the machine learning models. In this study, we preselected candidate features feeding machine learning models based on their frequency occurring in the patient population, which does not consider correlations between features. The performance of machine learning models can be improved by removing duplicated features.

Fourth, we only considered diagnosis and procedural features and neglected many other important features including laboratory variables, medications, and vital signs, which may be related to SMM.

Fifth, the concepts assigned to patients were considered in an atemporal fashion. It can potentially boost identification performance to model temporal relation between concepts. Yet chronological information between concepts reflects how SMM develops and is handled. Such information would be useful for characterizing how at-risk patients were managed.

## Figures and Tables

**Table 1–:** Summary statistics of diagnosis and procedural concepts assigned to patients

Class	Statistiscs	# of Diagnoses Assigned	# of Procedures Assigned
SMM	Mean	31.3	22.7
	Median	27	20.5
	IQR	22	16
Non-SMM	Mean	9.7	8.1
	Median	8	8
	IQR	6	5

IQR: inter-quartile range

**Table 2–:** Summary statistics of patient demographics

Class	Age (years)		Race (%)	
SMM	Mean	28.1	White	259 (62.9%)
	Median	28	Black	99 (24.0%)
	Q1	23	Asian	13 (3.1%)
	Q3	33	Other	41 (10.0%)
No evidance of SMM	Mean	28.1	White	30,626 (67.4%)
	Median	28	Black	8,396 (18.5%)
	Q1	24	Asian	2,316 (5.1%)
	Q3	32	Other	4,108 (9.0%)

Q1: first quartile; Q3: third quartile

**Table 3–:** The most important concepts that are positively associated with SMM (p-values smaller than 10^−7^)

Rank	Concept Name	Type	OR (95% CI)	Patients with this concept	SMM Patients with this concept
1	Dependence on ventilator	Condition	1938.9 (388.3 – 35217.1)	16	15
2	Intubation, endotracheal, emergency procedure	Procedure	1461.1 (428 – 9151.5)t	25	23
3	Critical care, evaluation and management of the critically ill or critically injured patient; first 30–74 minutes	Procedure	1735.4 (1076.8 – 2969.8)	184	166
4	Acute respiratory failure	Condition	1272.3 (644 – 2892.8)	80	72
5	Ventilation assist and management, hospital inpatient/observation, initial day	Procedure	1232.5 (663.6 – 2557.3)	95	85
6	Trauma and postoperative pulmonary insufficiency	Condition	1007.1 (486.4 – 2444.6)	62	55
7	Disorders of fluid and electrolytes	Condition	685.4 (261.3 – 2351)	27	23
8	Systemic inflammatory response syndrome	Condition	561.3 (229.1 – 1682.8)	12pt	23
9	Acidosis	Condition	325.8 (201.4 – 544.4)	82	59
10	Sepsis	Condition	308.2 (162.5 – 617.8)	44	31
